# Engagement of ubiquitination and de-ubiquitination at rostral ventrolateral medulla in experimental brain death

**DOI:** 10.1186/1423-0127-19-48

**Published:** 2012-04-30

**Authors:** Carol HY Wu, Julie YH Chan, Jimmy Li-Jer Chou, Samuel HH Chan, Alice YW Chang

**Affiliations:** 1 Center for Translational Research in Biomedical Sciences, Kaohsiung Chang Gung Memorial Hospital, Kaohsiung, 83301,, Taiwan, Republic of China; 2 Institute of Biomedical Science, National Sun Yat-sen University, Kaohsiung, 80424,, Taiwan, Republic of China

## Abstract

**Background:**

Whereas brain death is a vitally important clinical phenomenon, our contemporary understanding on its underlying cellular mechanisms remains elusive. This study evaluated whether the ubiquitin-proteasome system (UPS) in the rostral ventrolateral medulla (RVLM), a neural substrate that our laboratory identified previously to be intimately related to brain death, is engaged in this fatal process.

**Methods:**

We performed proteomics, Western Blot, real-time PCR, ELISA and pharmacological experiments in conjunction with a clinically relevant experimental endotoxemia model of brain death based on intravenous administration of *Escherichia coli* lipopolysaccharide in adult male Sprague–Dawley rats.

**Results:**

Proteomics, Western blot and enzyme activity analyses demonstrated that polyubiquitination was preserved and de-ubiquitination by ubiquitin C-terminal hydrolase isozyme-L1 (UCH-L1) was sustained, alongside increased monoubiquitin availability or proteasome activity in RVLM over the course of experimental endotoxemia. However, real-time PCR revealed no significant alteration in proteasome subunit alpha type-1, ubiquitin or UCH-L1 at mRNA level. Functionally, whereas microinjection into the bilateral RVLM of proteasome inhibitors (lactacystin or proteasome inhibitor II) potentiated survival, an inhibitor of ubiquitin-recycling (ubiquitin aldehyde) or an UCH-L1 inhibitor exacerbated mortality.

**Conclusions:**

We proposed previously that the progression towards brain death entails a tug-of-war between pro-death and pro-life programs in RVLM. It is conceivable that ubiquitination or de-ubiquitination in RVLM participate in brain death by regulating the degradation of the proteins involved in those programs.

## Background

Brain death is a clinical condition in which brain functions are demonstrated to be irreversibly absent [1]. Despite its paramount importance as a medical phenomenon, there is a dearth of information on its mechanistic underpinnings. Based on a computer algorithm that our laboratory developed for online and real-time spectral analysis of systemic arterial blood pressure (SAP) signals [2], we identified previously in a series of clinical studies that a common denominator exists in comatose patients who succumbed to systemic inflammatory response syndrome [3], organophosphate poisoning [4] or brain injury [5]. Death is invariably preceded by a dramatic reduction or loss in the low-frequency (LF) component (0.004 to 0.15 Hz) of the SAP spectrum. More importantly, whereas the LF spectral component is present in healthy subjects and patients in a persistent vegetative state, it is absent in patients diagnosed as brain dead [5]. Subsequent animal studies traced the origin of this clinical predictor of brain death to the rostral ventrolateral medulla (RVLM) in the brain stem [6]. We thus have in our hands a suitable neural substrate for the delineation of the cellular mechanisms that underpin brain death [7].

It is now clear that degradation of cellular proteins engages a highly complex, temporally controlled, and tightly regulated process that plays a major role in a variety of cellular processes during life and death as well as health and disease [8]. Most proteins in the cytosol and nucleus of eukaryotic cells are degraded via the ubiquitin-proteasome system (UPS), in a process that is energy-dependent. The highly conserved 76 amino acid protein ubiquitin is best known for its role in targeting proteins for proteasomal degradation. The active sites of the proteasome are protected from the cellular environment in the interior of the barrel-shaped 20 S structure. Polyubiquitinated proteins are recognized by the regulatory 19 S complexes of the proteasome, which unfold the protein substrates and assist in their translocation through a narrow gate into the 20 S core where degradation takes place [8,9]. The ubiquitin chain is released from the target protein remnant after the degradative process and is disassembled by de-ubiquitinating enzymes that include the ubiquitin C-terminal hydrolases (UCHs) [10]. The UCHs are responsible for the removal of small peptide fragments from the ubiquitin chain to generate free monomeric ubiquitin [10,11]. Of the three known mammalian members of the UCH family, UCH isozyme-L1 (UCH-L1) is among the most abundantly present proteins in brain [12].

We reported in a recent proteomic study [13] that key members of the UPS are present in RVLM at both mRNA and protein levels. The present study assessed the hypothesis that both ubiquitination and de-ubiquitination at RVLM are engaged in brain death.

## Materials and methods

All experimental procedures carried out in this study have been approved by the Institutional Animal Care and Use Committee of the Kaohsiung Chang Gung Memorial Hospital (#96008), and were in compliance with the guidelines for animal care and use set forth by this committee.

### Animals

Adult, male Sprague–Dawley rats (206–248 g; n = 322) were purchased from the Experimental Animal Center of the National Applied Research Laboratories, Taiwan. They were housed in our Association for Assessment and Accreditation of Laboratory Animal Care International-accredited Center for Laboratory Animals under temperature control (24 ± 0.5°C) and 12-h light–dark cycle (lights on during 08:00–20:00). Standard laboratory rat chow and tap water were available ad libitum. Animals were allowed to acclimatize for at least 7 days prior to experimental manipulations.

### General preparation

Under an initial pentobarbital sodium anesthesia (50 mg kg^-1^, IP), the trachea was intubated and the right femoral artery and both femoral veins were cannulated. Animals received thereafter intravenous infusion of propofol (Zeneca, Macclesfield, UK) at 20 mg kg^-1^ h^-1^. This scheme provides satisfactory anesthetic maintenance while preserving the capacity of central cardiovascular regulation [14]. The head of animals was fixed to a stereotaxic headholder (Kopf, Tujunga, CA, USA), and body temperature was maintained at 37°C with a heating pad. Animals were allowed to breathe spontaneously with room air during the recording session.

### Experimental endotoxemia model of brain death

An experimental endotoxemia model of brain death [7] was used, employing intravenous administration of *Escherichia coli* lipopolysaccharide (LPS; serotype 0111:B4, Sigma-Aldrich, St. Louis, MO, USA) (15 mg˙kg^-1^) as the insult, with saline serving as the vehicle control in some experiments. Temporal changes in SAP recorded from the femoral artery were routinely followed for 240 min. The SAP signals were also subject simultaneously to on-line and real-time power spectral analysis [2]. We were particularly interested in the LF (0.25-0.8 Hz) component in the SAP spectrum because its power density represents the most crucial link between our animal model and clinical observations from patients who died of systemic inflammatory response syndrome [3]. As we reported previously [15-17], the LF spectral component underwent triphasic changes that composed of a reduction (Phase I), augmentation (Phase II) and disappearance (Phase III) of its power density. Of note is that the last phase resembles that observed in our brain dead patients [5].

### Index for mortality

We assessed mortality by constructing a survival curve over 240 min after intravenous administration of LPS. Animals that succumbed to experimental endotoxemia exhibited a dramatic reduction of loss in the power density of the LF component of SAP signals before death [15-17].

### Collection of tissue samples from RVLM

We routinely collected tissue samples from RVLM [15-17] at the peak of each phase of experimental brain death. Medullary tissues collected from anesthetized animals but without treatment served as sham-controls. Tissues from both sides of the ventrolateral medulla, at the level of RVLM (0.5 to 1.5 mm rostral to the obex), were collected by micropunches made with a 1 mm (id) stainless steel bore to cover the anatomical boundaries of RVLM. The concentration of total proteins extracted was determined by the BCA Protein Assay (Pierce, Rockford, IL, USA).

### Sample preparation for proteomic and western blot analysis

As in previous studies [13,18,19], samples from RVLM were mixed with a tissue protein extraction reagent that contains protease inhibitor and T-PER (Roche, Basel, Switzerland). After being homogenized on ice, the mixture was centrifuged at 10,000 rpm. The supernatant was concentrated by precipitation overnight at −20°C using 10% trichloroacetic acid (TCA) and 0.1% dithiothreitol (DTT), followed by centrifugation at 13,000 rpm at 4°C. The pellet was air-dried after being washed twice with ice-cold acetone, and was reconstituted with a rehydration solution (2% 3-[(3-cholamidopropyl)-dimethylammonio]-1-propanesulfonate, 0.5% immobilized pH gradient (IPG) buffer, 8 M urea, 15 mM DTT and trace bromophenol blue).

### 2-Dimensional electrophoresis (2-DE)

The methods for 2-DE in a recent study [13] were used. In brief, isoelectric focusing (IEF) in the first dimension was carried out with immobiline DryStrip gels (13 cm in length, linear pH gradient 3–10; GE Healthcare, Piscataway, NJ, USA). The gels were rehydrated for 16 h with 300 μL rehydration solution covered by mineral oil, with the strips placed gel-side-down in an IPGphor strip holder (Amersham Pharmacia Biotech, Piscataway, NJ, USA). An IPGphor Isoelectric Focusing System (Amersham Pharmacia Biotech) was used for IEF, which was carried out at 20°C. A three-phase program was used for both analytical and preparative gels. The first phase was set at 500 V for 1 h, the second at 1,000 V for 1 h, and the third at a linear gradient spanning from 8,000 V to 16,000 V for 2 h. After the IEF run, the IPG gel strips were kept at −80°C or prepared directly for second-dimension electrophoresis.

The IPG gel strips were incubated at room temperature prior to second-dimension electrophoresis in a sodium dodecyl sulfate (SDS) equilibration solution (50 mM Tris–HCl, pH 8.8, 6 M urea, 2% SDS, 30% glycerol, and trace bromophenol blue) that contains 1% DTT; to be followed by incubation in SDS equilibration solution that contains 2.5% iodoacetamide. Second dimension run on the gels was carried out at 4°C on running SDS-PAGE gels (16 x 15 cm) without stacking using a Hoefer SE 600 (Amersham Pharmacia Biotech), and the IPG gel strips were embedded on top of the gels with 1% agarose. Electrophoresis was performed at 30 mA/gel for 5 h until bromophenol blue reached the bottom of the gel, after which the 2-D gels were stained with either Coomassie blue or silver nitrate.

The silver-stained 2-D gels were scanned in an ImageScanner II (Amersham Pharmacia Biotech), and the images were processed using Adobe Photoshop and PowerPoint software. Protein spots were checked manually to eliminate poorly detectable spots or artifacts due to gel distortion during silver staining, after which they were quantified and numbered using ImageMaster 2D Platinum (GE Healthcare). For this purpose, the intensity of each detected spot was first determined by ImageMaster, followed by computation of the area at 75% of the spot intensity. The volume of each spot in the 2-D gel was calculated based on these two parameters, and the relative volume of each spot was expressed as a percentage of the total volume of all quantified spots.

### In-gel digestion and MALDI-TOF mass spectrometry

As in a recent proteomics study [13], protein spots excised from the Coomassie blue-stained gels were destained with 0.2 ml acetonitrile and dried in a centrifugal evaporator. After rehydration in 10 mM DTT, alkylated with 100 mM iodoacetamide, the dried gels were digested on ice with digestion buffer made of 0.02 mg/ml trypsin gold (mass spectrometry grade; Promega, Madison, WI, USA) and 50 mM NH_4_HCO_3_. After removing excess solution, proteins were further digested for 15 h at 37°C. Peptides extracted with 50% acetonitrile in 5% formic acid were desalted and concentrated using in-tip reversed-phase resin (Zip Tip C18; Millipore, Billerica, MA, USA). Peptide mixtures were applied to the sample target and air-dried after being eluted from the Zip Tip with 0.1% trifluoroacetic acid (TFA) in 50% acetonitrile. After mixing with the matrix (α-cyano-4-hydroxycinnamic acid dissolved in 50% acetonitrile, 0.1% TFA), the sample was analyzed in a MALDI-TOF mass spectrometer system (Voyager DE-PRO, Applied Biosystems, Foster City, CA, USA) [13,18,19].

We measured the peptide masses as mono-isotopic masses. Peptide mass fingerprinting was searched against the NCBI database using MASCOT 2.1 (Matrix Science, Boston, MA, USA). Settings in the algorithm included *Rattus* as taxonomy, trypsin as enzyme, maximum of one missed cleavage site and assuming carbamidomethyl as a fixed modification of cysteine and oxidized methionine as a variable modification. Mass tolerance was set to 100 ppm.

### Western blot analysis

Western blot analysis [13,15-19] was carried out using a rabbit polyclonal antiserum against UCH-L1 (Santa Cruz, Santa Cruz, CA, USA); or a mouse monoclonal antiserum against ubiquitin (Santa Cruz) or β-actin (Chemicon, Temecula, CA, USA). This was followed by incubation with horseradish peroxidase-conjugated donkey anti-rabbit IgG (Amersham Biosciences, Little Chalfont, Bucks, UK) for UCH-L1; or sheep anti-mouse IgG (Amersham Biosciences) for ubiquitin or β-actin. Specific antibody-antigen complex was detected by an enhanced chemiluminescence Western blot detection system (Santa Cruz). The amount of protein was quantified by the ImageMaster Video Documentation System software (Amersham Biosciences), and was expressed as the ratio relative to β-actin protein.

### Isolation of RNA and real-time polymerase chain reaction (PCR)

Total RNA was isolated with TRIzol reagent (Invitrogen, Carlsgad, CA, USA). All RNA isolated was quantified by spectrophotometry and the optical density 260/280 nm ratio was determined. As in our previous studies [13,16], reverse transcriptase reaction was performed using a SuperScript Preamplification System (Invitrogen) for the first-strand cDNA synthesis. Real-time PCR analysis was performed by amplification of cDNA using a LightCycler® (Roche). PCR reaction for each sample was carried out in duplicate for all the cDNA and for the GAPDH control. Primers were designed using the sequence information of the NCBI database by Roche LightCycler® probe design software 2.0, and oligonucleotides were synthesized by Genemed Biotechnologies (Taipei, Taiwan).

The primer pairs used for amplification of target genes were:

DTT, Dithiothreitol; IEF, Isoelectric focusing; IκB, Inhibitory-κB; IPG, Immobilized pH gradient; LF component, Low-frequency component; LPS, Lipopolysaccharide; NF-κB, Nuclear factor-κB; NOS I, Nitric oxide synthase I; NOS II, Nitric oxide synthase II; RVLM, Rostral ventrolateral medulla; SAP, Systemic arterial blood pressure; SDS, Sodium dodecyl sulfate; TCA, Trichloroacetic acid; TFA, Trifluoroacetic acid; UCHs, Ubiquitin C-terminal hydrolases; UCH-L1, Ubiquitin C-terminal hydrolase isozyme-L1; UPS, Ubiquitin-proteasome system; psma-1, 5’-GTTGTAACCTCGCCGGA-3’ (forward primer) 5’-CTGCATGTGTCTTCGACTT-3’ (reverse primer); ubb, 5’-CGCACCCTCTCTGATTACA-3’ (forward primer) and 5’-CAAAGATGAGCCTCTGCTG-3’ (reverse primer); uch-l1, 5’-AAACGGAGAAGTTGTCCC-3’ (forward primer) and 5’-CGTCCACATTATTGAACAGGATAAA-3’ (reverse primer); gapdh, 5’-CTTCTCTTGTGACAAAGTGGAC-3’ (forward primer) 5’-TTAGCGGGATCTCGCTC-3’ (reverse primer).

Fluorescence signals from the amplified products were quantitatively assessed using the LightCycler® software program (version 3.5; Roche). Second derivative maximum mode was chosen with baseline adjustment set in the arithmetic mode. The relative changes in mRNA expression were determined by the fold-change analysis in which

(1) $$\text{Fold change}=2^{-[\Delta \Delta Ct]},\text{where}\Delta \Delta Ct=(Ct_{gene}-Ct_{GAPDH}){}_{\text{L}\text{P}\text{S}\text{treatment}}-(Ct_{gene}-Ct_{GAPDH}){}_{\text{s}\text{h}\text{a}\text{m}\text{c}\text{o}\text{n}\text{t}\text{r}\text{o}\text{l}}).$$

Note that Ct value is the cycle number at which fluorescence signal crosses the threshold.

### Proteasome activity

We measured proteasome activity with a commercial kit **(**Proteasome-Glo 3-Substrate System; Promega, Madison, WI) according to the recommended protocol. Proteins extracted from the ventrolateral medulla were incubated with proteasome-Glo reagent at room temperature for 2 h. For the purpose of the present study, we used the luminogenic substrates provided for the detection of chymotrypsin-like activity, Suc-LLVY-aminoluciferin; trypsin-like activity, Z-LRR-aminoluciferin and caspase-like activity, Z-nLPnLD-aminoluciferin. Proteasome activity was measured with a luminometer (Berthold Technologies, Bad Wildbad, Germany) and expressed as fold changes against sham-controls.

### Microinjection of test agents into RVLM

Test agents were microinjected bilaterally and sequentially into RVLM via a glass micropipette connected to a 0.5-μL Hamilton (Reno, NV, USA) microsyringe [15-19]. The coordinates used were: 4.5 to 5 mm posterior to the lambda, 1.8 to 2.1 mm lateral to the midline and 8.1 to 8.4 mm below the dorsal surface of the cerebellum. As a routine, a total volume of 50 nl was delivered to each side of RVLM over 2–3 min to allow for complete diffusion of the test agents to an area approximately 800 x 800 μm within the anatomical boundaries of RVLM [18]. Test agents used included a non-selective proteasome inhibitor [20], lactacystin (Calbiochem, San Diego, CA, USA); a specific inhibitor of chymotrypsin-like proteasomal activity [21], proteasome inhibitor II (Calbiochem); a general inhibitor of ubiquitin-recycling [22], ubiquitin aldehyde (Calbiochem); and a potent, reversible, competitive and active site-directed inhibitor of UCH-L1 [23], UCH-L1 inhibitor (Calbiochem).The doses were adopted from previous reports that used those test agents for the same purpose as in this study. Lactacystin or ubiquitin aldehyde was dissolved in artificial cerebrospinal fluid, and proteasome inhibitor II or UCH-L1 inhibitor was dissolved in 10% and 40% DMSO respectively. Possible volume effect of microinjection was controlled by injecting the same amount of solvent. All test agents or their vehicles were given 30 min before LPS administration. To avoid the confounding effects of drug interactions, each animal received only one test agent.

### Statistical analysis

All values are expressed as mean ± SEM. Changes in protein expression, real-time PCR products or enzyme activity in RVLM during each phase of experimental brain death was used for statistical analysis. One-way ANOVA was used to assess group means. This was followed by the Scheffé multiple-range test for post hoc assessment of individual means. Mortality rate was assessed by the Fisher exact test. *P <*0.05 was considered to be statistically significant.

## Results

### Members of the UPS are present in the proteome of RVLM

**Figure 1:**
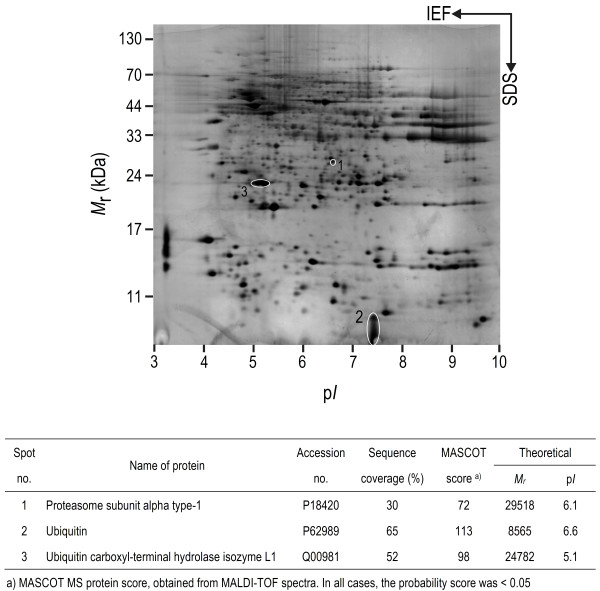
**Members of the UPS are present in the proteomes of RVLM.** Representative silver-stained 2-D electrophoresis gel of the rostral ventrolateral medulla (RVLM) showing the location of the protein spots studied. Results are representative of 22 individual samples from 22 animals. 1, proteasome subunit alpha type-1; 2, ubiquitin; 3, ubiquitin carboxyl-terminal hydrolase isozyme L1 (UCH-L1). Shown also is the identification of those proteins by MALDI-TOF analysis in association with the MASCOT search program.

**Figure 2:**
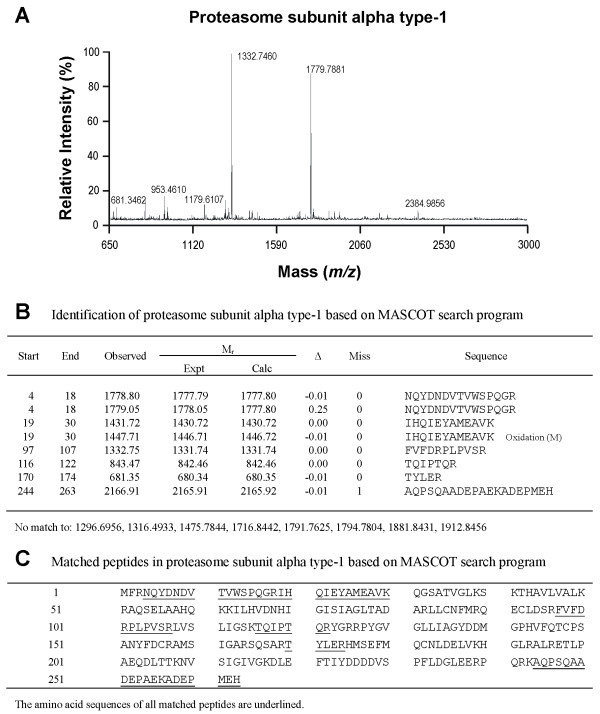
**Identification of proteasome subunit alpha type-1.** Tryptic peptide spectrum of proteasome subunit alpha type-1 generated by MALDI-TOF mass spectrometry (**A**); and identification of proteasome subunit alpha type-1 based on the MASCOT search program (**B,C**).

**Figure 3:**
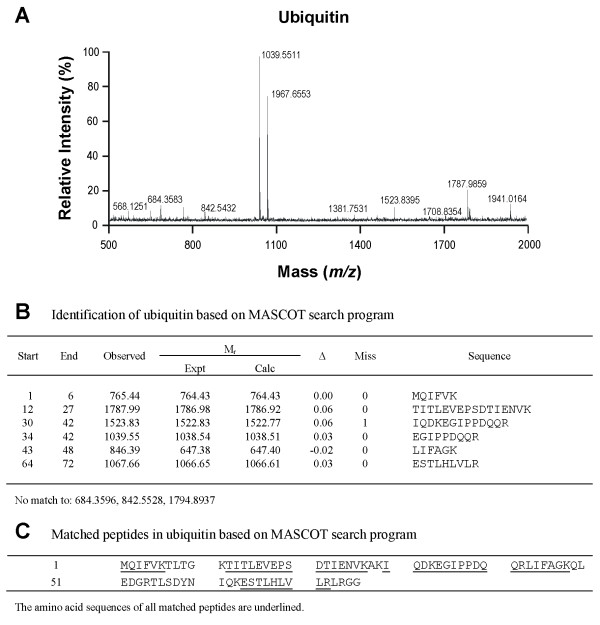
**Identification of ubiquitin.** Tryptic peptide spectrum of ubiquitin generated by MALDI-TOF mass spectrometry (**A**); and identification of ubiquitin based on the MASCOT search program (**B,C**).

**Figure 4:**
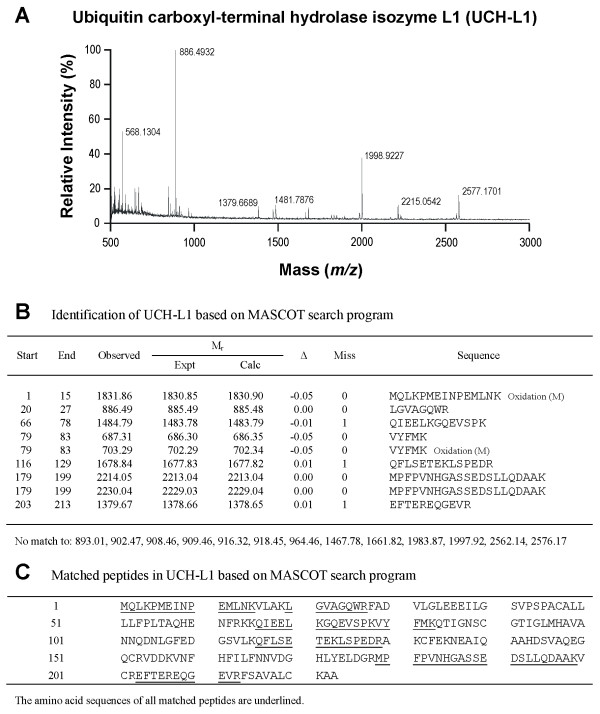
**Identification of UCH-L1.** Tryptic peptide spectrum of UCH-L1 generated by MALDI-TOF mass spectrometry (**A**); and identification of UCH-L1 based on the MASCOT search program (**B,C**).

As in our recent study [13], silver-stained 2-D electrophoresis gels reproducibly resolved 699 ± 29 (n = 22) protein spots in the domain of pI: 3–10 and Mr: 11–130 kDa from RVLM (Figure 1). Based on MALDI-TOF mass spectrometric analysis on protein spots in the Coomassie blue-stained 2-D electrophoresis gels from randomly selected samples (n = 8), in conjunction with search results using the MASCOT program (Figures 1,2,3 and 4) we found that members of the UPS, including proteasome subunit alpha type-1, ubiquitin and UCH-L1 are present in RVLM of rats that were subject to experimental brain death and the sham-controls.

### Ubiquitination in RVLM remains operational during experimental brain death

**Figure 5:**
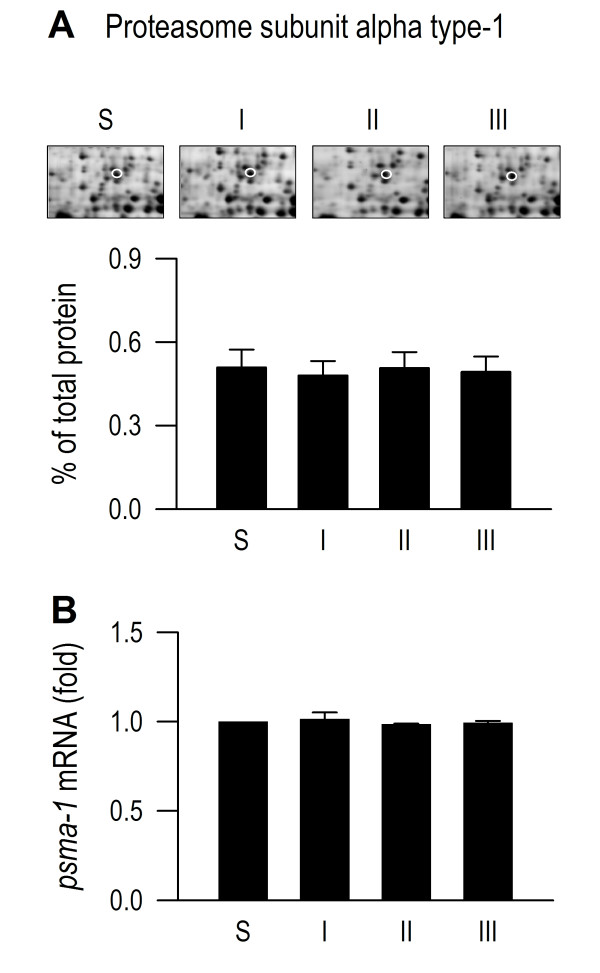
**Ubiquitination in RVLM remains operational during experimental brain death.** Phasic changes in spot volume of proteasome subunit alpha type-1 in the proteomic map of RVLM (**A**) or fold changes against sham-controls of *psma-1* mRNA determined by real-time PCR (**B**) in RVLM of rats that received intravenous administration of LPS (15 mg kg^-1^). In this and Figures 6, 8 and 9, S denotes samples obtained from sham-controls, and I, II or III denotes samples obtained during the peak of Phases I, II or III in our experimental endotoxemia model of brain death. Values are mean ± SEM, n = 5–6 animals per experimental group. No statistical significance (P > 0.05) found among all groups in one-way ANOVA.

**Figure 6:**
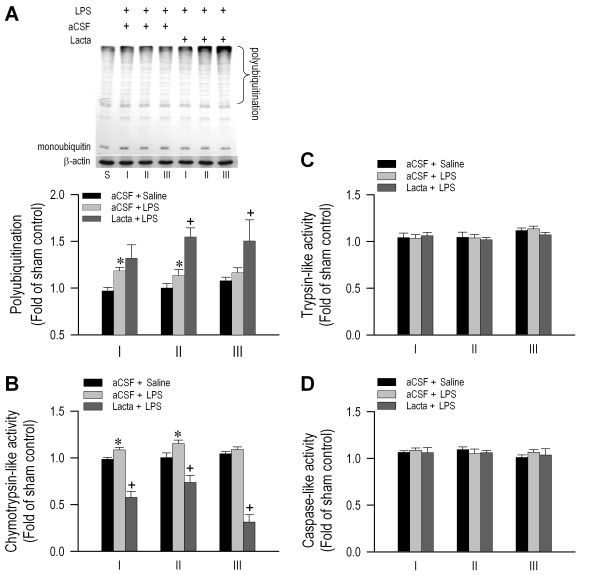
**Maintained polyubiquitination and preserved proteasomal activity in RVLM during experimental brain death.** Illustrative gels or summary of phasic fold changes against sham-controls in polyubiquitinated proteins detected by Western blot analysis (**A**) or chemotrypsin-like (**B**), trypsin-like (**C**) and caspase-like (**D**) proteasome activity in RVLM of rats that received pretreatment by microinjection bilaterally into RVLM of lactacystin (Lacta; 1 nmol) or artificial cerebrospinal fluid (aCSF), followed by intravenous administration of LPS (15 mg kg^-1^) or saline. Values are mean ± SEM, n = 5–6 animals per experimental group. *P < 0.05 versus aCSF + Saline group, and ^+^P < 0.05 versus aCSF + LPS group at corresponding phases in the Scheffé multiple-range test.

Our first series of experiments addressed the fundamental issue of whether ubiquitination in RVLM remains operational during experimental brain death. Proteomic analysis (Figure 5A) revealed that the expression of proteasome subunit alpha type-1 remained stable throughout the three phases of our experimental endotoxemia model, in association with intravenous administration of LPS (15 mg kg^-1^). These observations were confirmed by the insignificant phasic changes in *psma-1* mRNA as determined by real-time PCR (Figure 5B). Western blot analysis further showed that polyubiquitination in RVLM remained prevalent (Figure 6A). Moreover, the degree of polyubiquitination was augmented by pretreatment with microinjection into the bilateral RVLM of a non-selective proteasome inhibitor [20], lactacystin (1 nmol), a conventional index for preserved proteasomal activity. Measurement of enzyme activity (Figure 6B) additionally revealed that the chemotrypsin-like proteasome activity in RVLM was enhanced during experimental brain death, to be antagonized by pretreatment with lactacystin (1 nmol). On the other hand, both trypsin-like and caspase-like activities were essentially unaltered, and were unaffected by lactacystin pretreatment (Figure 6C,D).

### Ubiquitination in RVLM precipitates fatality during experimental brain death

**Figure 7:**
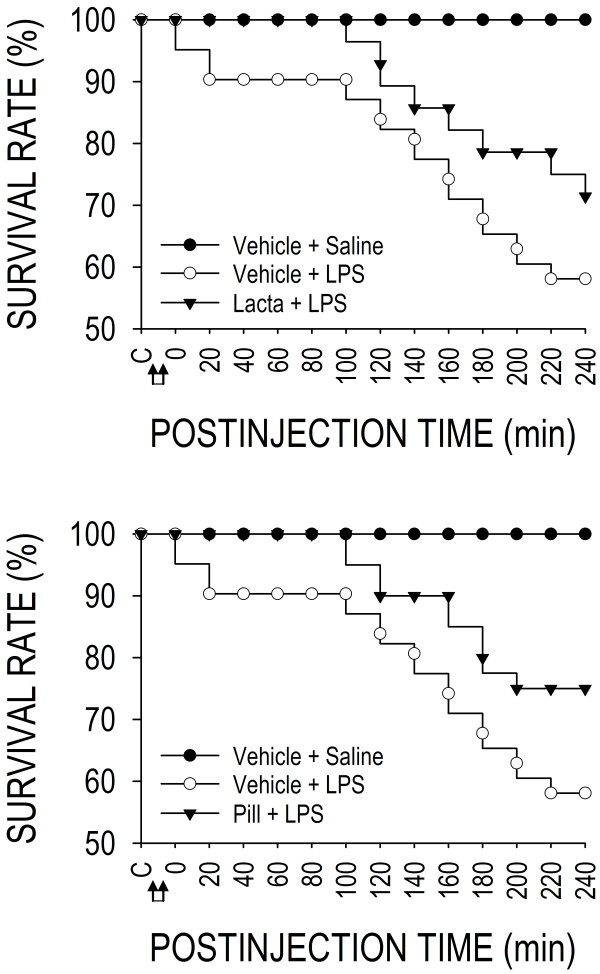
**Ubiquitination in RVLM precipitates fatality during experimental brain death.** Survival rate of rats that received pretreatment by microinjection bilaterally into RVLM of lactacystin (Lacta; 1 nmol) (upper diagram), proteasome inhibitor II (PiII; 1 nmol) (lower diagram) or their solvents (Vehicle), followed by intravenous administration (at arrows) of LPS (15 mg kg^-1^) or saline. Values are mean ± SEM, n = 7–8 animals at the beginning of the experiment. Significant difference versus Vehicle + LPS group in the Fisher exact test.

Our second series of experiments delineated the functional significance of the operational ubiquitination in RVLM during experimental brain death. Figure 7 illustrated that, given at the dose we used (15 mg kg^-1^), intravenous administration of LPS induced a progressive increase in the number of animals that succumbed to endotoxemia beginning 100 min after administration of LPS, reaching a mortality rate of approximately 40% within 240 min. Intriguingly, inhibiting proteasome activities by microinjection bilaterally of lactacystin (1 nmol; Figure 7, upper diagram) or a specific inhibitor of chymotrypsin-like proteasomal activity [21], proteasome inhibitor II (1 nmol; Figure 7, lower diagram) into RVLM significantly improved the 4-h survival rate to 70-75%.

### De-ubiquitination in RVLM also remains operational during experimental brain death

**Figure 8:**
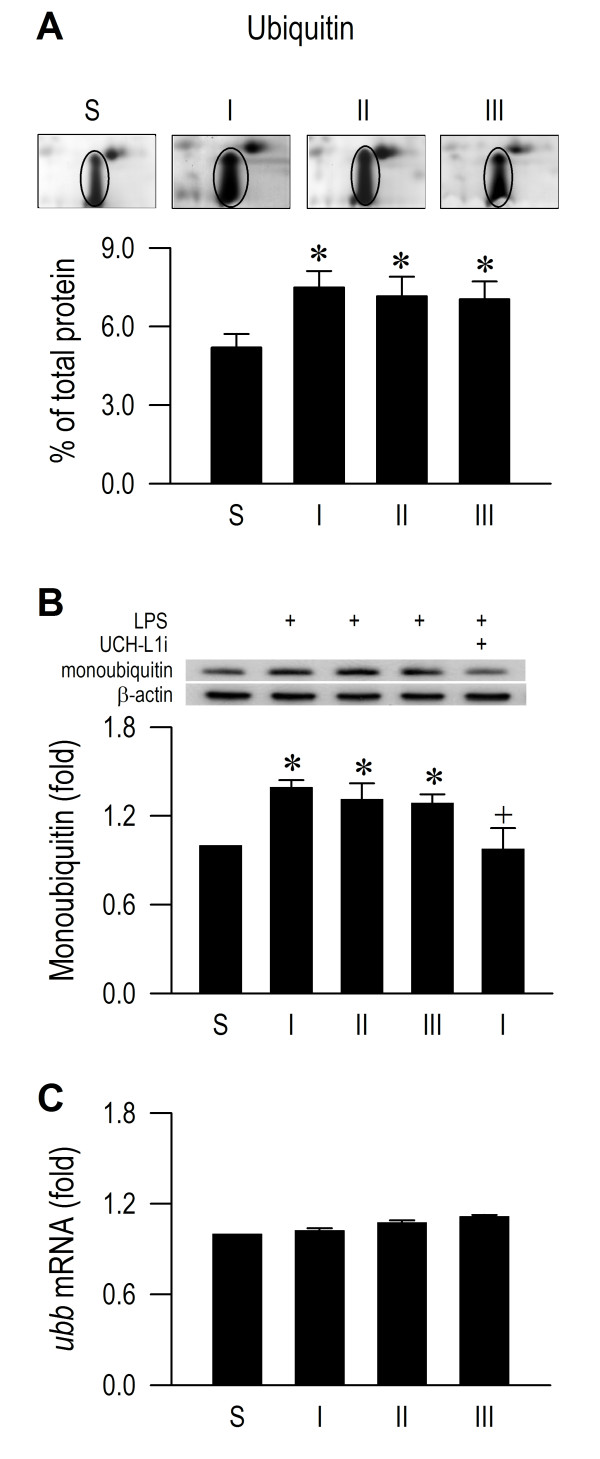
**De-ubiquitination in RVLM also remains operational during experimental brain death.** Phasic changes in spot volume of ubiquitin in the proteomic map of RVLM (**A**); fold changes against sham-controls of monoubiquitin detected by Western blot analysis (**B**) or *ubb* mRNA determined by real-time PCR (**C**) in RVLM of rats that received intravenous administration of LPS (15 mg kg^-1^), alone or with additional pretreatment by microinjection into bilateral RVLM of UCH-L1 inhibitor (UCH-L1i; 1 nmol). Values are mean ± SEM, n = 5–6 animals per experimental group. *P < 0.05 versus sham-control group, and ^+^P < 0.05 versus LPS group at corresponding phase in the Scheffé multiple-range test.

**Figure 9:**
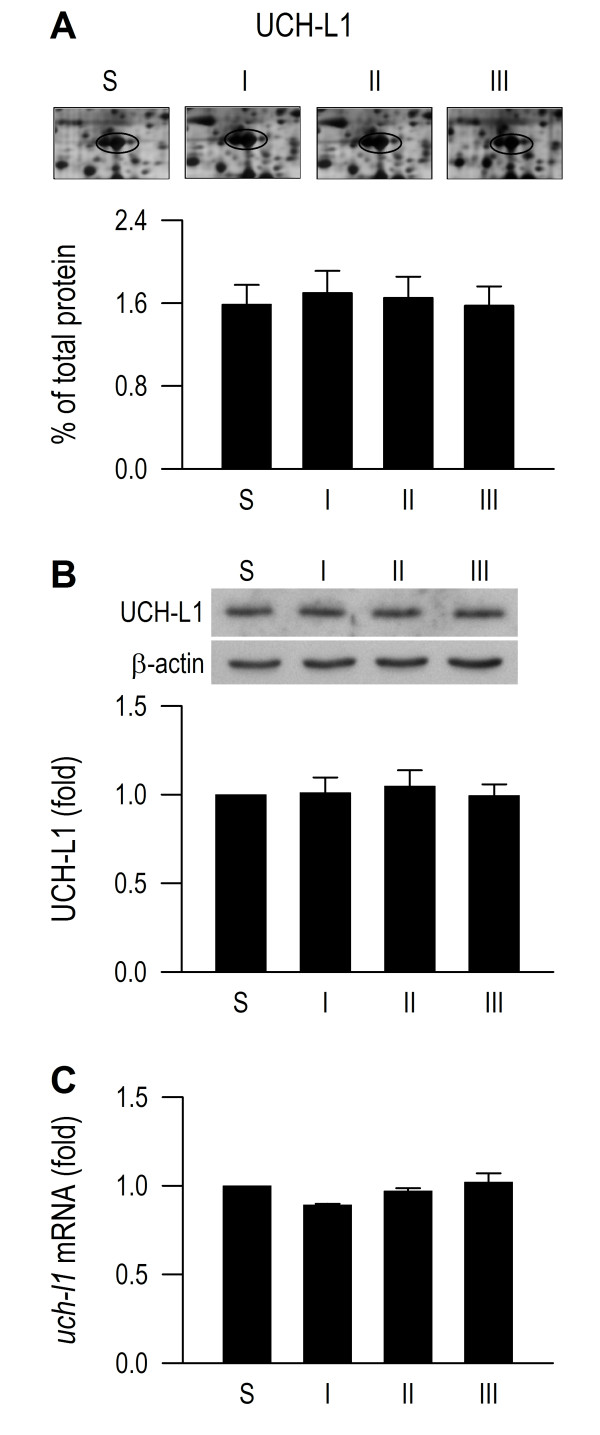
**Preserved UCH-L1 functionality despite lack of synthesis.** Phasic changes in spot volume of UCH-L1 in the proteomic map of RVLM (**A**); fold changes against sham-controls of UCH-L1 detected by Western blot analysis (**B**) or *uch-l1* mRNA determined by real-time PCR (**C**) in RVLM of rats that received intravenous administration of LPS (15 mg kg^-1^). Values are mean ± SEM, n = 5–6 animals per experimental group. No statistical significance (P > 0.05) found among all groups in one-way ANOVA.

We next investigated whether de-ubiquitination, which avails monoubiquitin (free form of ubiquitin) for optimal operation of polyubiquitination in RVLM also remains functional during experimental brain death. We found that whereas the spot volume of ubiquitin in the 2-D electrophoresis gels (Figure 8A) and monoubiquitin as determined by Western blot analysis (Figure 8B) were significantly elevated in RVLM in our experimental endotoxemia model, the mRNA level of ubiquitin remained constant (Figure 8C). Likewise, superimposed on insignificantly altered protein (Figure 9A,B) and mRNA (Figure 9C) levels, UCH-L1 functionality was preserved because pretreatment with a specific UCH-L1 inhibitor (1 nmol) [23] significantly antagonized the elevated monoubiquitin level (Figure 8B).

### De-ubiquitination is crucially involved in maintaining survival during experimental brain death

**Figure 10:**
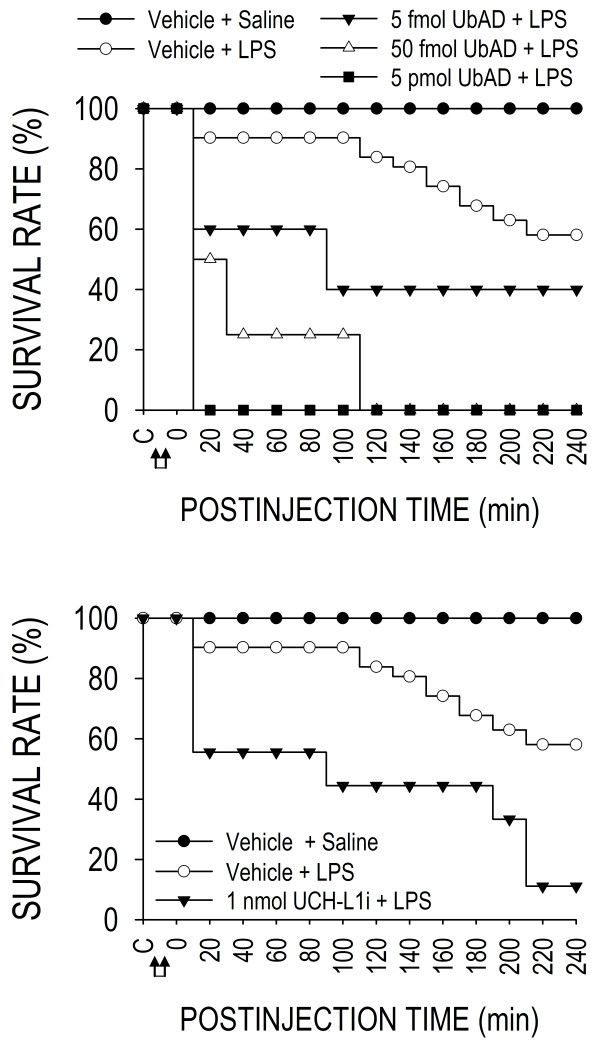
**De-ubiqutination is crucially involved in maintaining survival during experimental brain death.** Survival rate of rats that received pretreatment by microinjection bilaterally into RVLM of ubiquitin aldehyde (UbAD; upper diagram), UCH-L1 inhibitor (UCH-L1i; lower diagram) or their solvents (Vehicle), followed by intravenous administration (at arrows) of LPS (15 mg kg^-1^) or saline. Values are mean ± SEM, n = 7–8 animals at the beginning of the experiment. Significant difference versus Vehicle + LPS group in the Fisher exact test.

Our final series of experiments delineated the functional significance of de-ubiquitination in RVLM during experimental brain death. Pretreatment by microinjection bilaterally into RVLM of a general inhibitor of ubiquitin-recycling [22], ubiquitin aldehyde, at a very low dose of 5 fmol, was effective in reducing the survival rate by 40% within 10 min after the initiation of experimental endotoxemia, reaching 60% mortality by 80 min (Figure 10, upper diagram). Increasing the dose to 50 fmol exacerbated the mortality rate to 80% by 20 min, with all animals succumbed to endotoxemia by 110 min. Pretreatment with ubiquitin aldehyde at 5 pmol resulted in 100% mortality within 5–10 min after the administration of LPS. Specific inhibition of UCH-LI (1 nmole) in RVLM also significantly reduced survival rate that began within the first 20 min in animals subject to experimental endotoxemia, leading to a survival rate of 10% by the end of 240 min (Figure 10, lower diagram).

## Discussion

The UPS has emerged in recent years as a central player under physiological and pathological conditions in the regulation of diverse cellular processes. In addition to degradation of abnormal proteins, the UPS is responsible for the recognition and degradation of ubiquitinated substrate such as proteins involved in cell cycle regulation, transcriptional regulation, receptor function, signal transduction, endocytosis, antigen presentation, aging, stress responses or apoptosis [8,9,24,25]. Aberrations in either the process of ubiquitination or de-ubiquitination have also been directly implicated in the etiology of many diseases [8,9], including cardiomyopathy [26]. Based on an endotoxemia model and in conjunction with proteomics, Western blot, real-time PCR, enzyme activity and survival evaluations, the present study revealed that augmented polyubiquitination, enhanced proteasomal activities and recycling of ubiquitin through sustained de-ubiquitination in RVLM play a vital role during brain death.

As reflected by Hershko [27], the discovery of the UPS was originally driven by the notion that intracellular protein degradation is energy-dependent. It is since known that two ATP-dependent steps are present during ubiquitination; formation of a high-energy thiolester bond between ubiquitin and the ubiquitin-activating enzyme and degradation of ubiquitinylated proteins by the 26 S proteasome complex [8,9]. In this context, it is intriguing to note that enzyme assay for activity in or electron transport capacity between respiratory chain complexes in our previously study [28] revealed that the progressive reduction in neuronal activity at RVLM in our experimental endotoxemia model of brain death is associated with bioenergetic failure because of mitochondrial dysfunction. An immediate corollary to those observations is that the process of polyubiquitination and proteasomal degradation in RVLM should be progressively compromised during brain death. On the contrary, we observed in the present study that despite minimal alterations in proteasome subunit alpha type 1 mRNA and protein, there was sustained polyubiquitination and enhanced proteasomal activity in RVLM during experimental brain death. By demonstrating that RVLM is a high metabolic energy-required region in brain, we speculated in a recent study [13] that “the presence of higher levels of tissue oxygen and ATP synthase subunits in RVLM, leading to augmented ATP production, provides a cellular safeguard mechanism to reduce the possibility of irreversible reduction in intracellular ATP contents that precipitate brain death”. Results from pretreatments with lactacystin or proteasome inhibitor II on survival in the present study, however, suggest that one of the targets of this cellular safeguard mechanism is polyubiquitination and proteasomal degradation of the pro-life proteins in RVLM during experimental brain death.

Optimal operation of polyubiquitination (conjugation of protein substrates by ubiquitin) also depends on the availability of monoubiquitin (free form of ubiquitin), which is regulated by the de-ubiquitinating enzymes [10]. By showing the effectiveness of ubiquitin aldehyde at fmol concentration to exacerbate mortality rate, the present study revealed that recycling of ubiquitin in RVLM is actively executed and is vitally involved in the maintenance of survival in our experimental endotoxemia model of brain death. Of the known de-ubiquitination enzymes, UCH-L1 is among the most abundantly present proteins in brain [12]. Our laboratory reported previously that UCH-L1 in RVLM is crucial to survival during intoxication induced by the organophosphate poison mevinphos [29]. The present study further showed that superimposed on minimal alterations in UCH-LI mRNA and protein, there is an enhanced availability in free ubiquitin that was suppressed by UCH-L1 inhibitor. Furthermore, on an equimolar basis (1 nmol), the degree of improvement of survival rate by lactacystin or proteasome inhibitor II was significantly less than the detrimental effects of UCH-L1 inhibitor. There are two potential interpretations on those results. First, despite the lack of de novo synthesis, the abundantly present UCH-L1 in RVLM may be sufficient to avail additional recycled monoubiquitin through active de-ubiquitination to sustain the elevated polyubiquitination. Second, the augmented polyubiquitination favors maintenance of survival by promoting the degradation of pro-death proteins in RVLM during experimental brain death. Further studies are required to delineate the mechanisms via which the de-ubiquitination processes and subsequent polyubiquitination may target preferentially the pro-death proteins.

Our laboratory proposed previously [7] that the fundamental conceptual backbone in the pursuit of the cellular and molecular mechanisms that underlie brain death is that the progression towards death entails multiple tug-of-wars between pro-death and pro-life programs in RVLM [7]. It is therefore conceivable that ubiquitination or de-ubiquitination in RVLM participate in brain death by regulating the degradation of the proteins involved in those programs. One of the best-known targets of the UPS is activation of the inducible transcription factor nuclear factor-κB (NF-κB) [30,31]. NF-κB is retained in a latent form in the cytoplasm of non-stimulated cells by inhibitory molecules collectively termed inhibitory-κB (IκB). Stimuli that induce NF-κB activation target IκB to site-specific phosphorylation, leading to its degradation by the UPS. Following IκB degradation, NF-κB is translocated to the nucleus as an active transcription factor that is able to induce its target genes. It is thus of interest that, based on an experimental endotoxemia model, our laboratory found previously [15,17,32] that the advancement towards brain death is associated with the progressive augmentation in both molecular synthesis and functional expression of nitric oxide synthase II (NOS II) in RVLM. We further showed that transcriptional regulation by NF-κB is crucial to the expression of NOS II gene [15]. At the same time, a number of reports suggest that the UPS may also be involved in the degradation of NOS II [33,34]. It follows that the UPS may participate actively in brain death by regulating both the synthesis (via activation of NF-κB through IκB degradation) and degradation of NOS II in RVLM. Our laboratory demonstrated recently [35] that the UPS at RVLM indeed plays such a double-edged sword role in brain death. Specifically, we showed that a crucial determinant for the prevalence of NOS II in RVLM during brain death resides in the temporal balance between its continuous degradation and the progressively augmented synthesis. In concordance with findings in the present study, we further found that recycling of ubiquitin in RVLM through sustained de-ubiquitination is crucial to uninterrupted degradation of NOS II, which is essential for the maintenance of survival. It should be mentioned that in addition to the UPS, other signaling systems in RVLM are also engaged in experimental brain death. For example, the molecular synthesis of NOS I in RVLM, which is crucial to the pro-life process [15,18,32], is downstream to the hypoxia-inducible factor-1/heme oxygenase-1/heat shock protein 70 cascade [18,36]; and hypoxia-inducible factor-1 is stabilized by sumoylation [37].

## Conclusion

In conclusion, based on an experimental endotoxemia model, the present study provided novel findings to support the notion that both ubiquitination and de-ubiquitination in RVLM participate in brain death. We found that polyubiquitination was augmented, proteasomal activities were enhanced and recycling of ubiquitin was sustained through de-ubiquitination in RVLM during experimental brain death. We stipulate that the UPS is engaged in the multiple tug-of-wars between pro-death and pro-life programs in RVLM during the progression towards brain death by regulating the degradation of the proteins involved. This information will offer important insights into the etiology of brain death and shall be invaluable to future development of management strategies against this fatal eventuality.

## Abbreviation

DTT, Dithiothreitol; IEF, Isoelectric focusing; IκB, Inhibitory-κB; IPG, Immobilized pH gradient; LF component, Low-frequency component; LPS, Lipopolysaccharide; NF-κB, Nuclear factor-κB; NOS I, Nitric oxide synthase I; NOS II, Nitric oxide synthase II; RVLM, Rostral ventrolateral medulla; SAP, Systemic arterial blood pressure; SDS, Sodium dodecyl sulfate; TCA, Trichloroacetic acid; TFA, Trifluoroacetic acid; UCHs, Ubiquitin C-terminal hydrolases; UCH-L1, Ubiquitin C-terminal hydrolase isozyme-L1; UPS, Ubiquitin-proteasome system; psma-1, 5’-GTTGTAACCTCGCCGGA-3’ (forward primer) 5’-CTGCATGTGTCTTCGACTT-3’ (reverse primer); ubb, 5’-CGCACCCTCTCTGATTACA-3’ (forward primer) and 5’-CAAAGATGAGCCTCTGCTG-3’ (reverse primer); uch-l1, 5’-AAACGGAGAAGTTGTCCC-3’ (forward primer) and 5’-CGTCCACATTATTGAACAGGATAAA-3’ (reverse primer); gapdh, 5’-CTTCTCTTGTGACAAAGTGGAC-3’ (forward primer) 5’-TTAGCGGGATCTCGCTC-3’ (reverse primer)..

## Competing interests

The authors declare that they have no competing financial interests.

## Authors’ contributions

CHYW performed the experiments. JLJC performed the proteomic experiments. JYHC participated in experimental design. SHHC and AYWC conceived the study, participated in experimental design, and drafted and revised the manuscript. All authors have read and approved the final manuscript.
